# Gold clay from self-assembly of 2D microscale nanosheets

**DOI:** 10.1038/s41467-019-14260-5

**Published:** 2020-01-29

**Authors:** Youfeng Yue, Yasuo Norikane

**Affiliations:** Electronics and Photonics Research Institute, National Institute of Advanced Industrial Science and Technology (AIST), Higashi 1-1-1, Tsukuba, Ibaraki 305-8565 Japan

**Keywords:** Two-dimensional materials, Self-assembly

## Abstract

Nature has always demonstrated incredible ability to create amazing materials such as soft clay which are built from nanoplatelet packing structures. It is challenging to produce artificial clays owing to the difficulty in obtaining large volume fractions of nanoplatelets and the lack of structural control in layer-by-layer packing. Here, single-crystalline Au nanosheets are synthesized by controlled growth in the bilayer membranes of succinic acid surfactants. Then, a self-assembly strategy is used to make {111}-oriented gold nanostructures at the liquid−liquid interface. The stiffness of the nanosheet assemblies are six orders of magnitude softer than bulk gold. The Au nanosheet aggregates show high plasticity and deformable into macroscale free-standing metallic architectures. They show a stress/strain-dependent conductivity owing to morphological changes. Our study provides valuable insights on the chemical synthesis of 2D nanostructures as well as for the self-assembly strategy on fabrication of mouldable metals for producing free-standing metallic architectures with microscale resolutions.

## Abstract (web summary)

Here, the authors report controlled growth of Au nanosheets on bilayer membranes and self-assembly of nanosheets leads to free-standing gold clay nanostructures. The gold nanostructures possess six orders of magnitude softer stiffness compared to bulk gold, higher plasticity and deformability.

## Introduction

Tremendous efforts have been made towards developing nano/microscale materials with well-defined shapes and properties using various fabrication techniques such as molecular self-assembly or seed-mediated methods^1–4^. However, scaling-up these procedures to produce free-standing and three-dimensional macroscopic materials remains a significant challenge^5^.

In nature, although clays are composed of nanomaterials, they can be easily modulated into macroscopic shapes. Nature has inspired researchers to produce artificial metal clays using nanomaterials, but thus far, studies reported in the literature on metal clays remain limited compared with the synthesis of nanomaterials. There has been one interesting report on mouldable gold clay based on the self-assembly of gold nanoparticles^6^. However, metal clays derived from nanoparticles are prone to collapsing or losing their structural detail, especially when they are hardened at temperatures above 300 °C^6^. Similarly, sea sand (spherical, diameter >74 μm) has enough attractive force to be made into a castle in the presence of water. But when the sand is dehydrated, such structures crumble under their own weight. These situations contrast with the formation of ceramics from natural clays, which can always hold their shapes, even during high-temperature calcining.

Different from the abovementioned nano/microscale particles, natural clays are mainly composed of 2D silicates with symmetric hexagonal shapes of microscale sizes (~2 μm)^7^. The microscale flat-shaped sheets interlace strongly with each other, even when dehydrated. Moreover, the surface areas and attractive forces (defined as the interactions between adsorbed molecules or ions and surface atoms of clay minerals) fasten the plates together^8^.

Inspired by this, here, we demonstrate a soft gold clay material consisting of 2D {111}-oriented microscale nanosheets. The Au nanosheets are synthesized by a method from dodecenylsuccinic acid (DSA) bilayers, which serve as a soft template for 2D controlled growth. The synthesized thin gold nanosheets can self-assemble into {111}-oriented films at the liquid−liquid interface. The nanostructure assemblies are soft, plastic, and deformable into high-resolution, free-standing complex metallic shapes at room temperature. Furthermore, their shape and structural detail are maintained upon mild and high-temperature thermal annealing, and they are highly electrically conductive in both plastic and deformed states. The {111}-oriented nanosheets as ordered arrays can also fuse into superstructures with a compact and smooth surface upon compressive force at room temperature. We found the electronic properties of these films are dependent on external forces (stress/strain). The film conductivity, for example, increased at least three orders of magnitude under a compression strain of 90%, with conductivity values comparable to that of bulk gold. All these findings provide valuable insights in the fundamental synthesis of 2D metallic nanostructures as well as for the self-assembly process on the fabrication of hybrid soft metals. The development of soft metals that can be produced into free-standing complex metallic structures with high resolution may open up new avenues for creating conductive electronics and devices.

## Results

### Synthesis of thin and microscale gold nanosheets

**Fig. 1:**
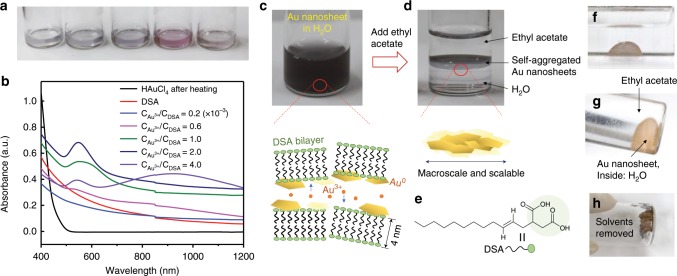
2D confined growth of gold nanosheets and their self-assembly at the liquid−liquid interface. **a** Photographs of as-prepared Au^0^ nanostructures synthesized in DSA aqueous solution (53 °C) for 20 min with addition of different Au^3+^ concentrations. From left to right C_Au_^3+^/C_DSA_ mole ratio: 0.2, 0.6, 1.0, 2.0, and 4.0 × 10^−3^. **b** Corresponding UV−Vis−NIR absorbance spectra of solutions. **c** Synthesized gold nanomaterials dispersed in water containing DSA bilayer structures. **d** Gold nanosheets self-assembled at the liquid−liquid interface. **e** Chemical structure of the DSA molecules. **f**, **g** The nanosheet assemblies prefer to exist at the liquid−liquid interface with a spherical shape. **h** The nanosheet assemblies show a brown colour after solvent removal.

The gold nanosheets were synthesized by using the method described in Fig. 1. DSA bilayer structures served as a 1D confined physical environment for controlled 2D growth of the nanosheets. In our previous work, we developed various bilayer structures in water, hydrogels, and films^9–15^. Here, the DSA bilayer structure was composed of 100−200-nm-thick water layers sandwiched by two bilayer membranes of self-assembled DSA surfactants. The DSA water solution (~1.1 wt.%) shows iridescent structural colours due to Bragg’s reflection of visible light on the periodic lamellar membranes. Selected amounts of chloroauric acid aqueous solution (HAuCl_4_·3H_2_O, 2 × 10^−3^ M) were subsequently introduced into the water layers. The systems contained excess DSA since the HAuCl_4_/DSA mole ratios were <0.006. Next, the mixtures were kept in a water bath at 53 °C without any stirring. A reaction first occurred at the water/air interface, where a colour appeared due to the localized surface plasmon resonance (LSPR), and then the colour spread to the bulk solution. Finally, the entirety of the water changed colour within 15 min, indicating the production of a large amount of gold nanomaterials (Supplementary Movie 1, Supplementary Fig. 1). Compared with the traditional method using citrate that requires a high temperature (e.g. 100 °C), strong stirring, and a long reaction time^16^, this method is beneficial for large-scale synthesis and practical applications. In this reaction, the Au^3+^ ions in the water diffuse along the planes of the DSA bilayer membranes (with a large amount of carboxylate groups) and grow in a preferred direction. The chemical structure of DSA is in fact somewhat similar to that of citrate, with the nearest two carboxylate groups separated by three carbon atoms. Yin et al. found that such structures prefer to bind to {111} facets of metal nuclei and thus stabilize continuous anisotropic growth^17^. In this system, we found that single carboxylate ligand-DSA bilayer membranes are sufficient for obtaining a spontaneous growth of gold nanomaterials without any additional reducing agents. We synthesized various DSA compounds with different alkyl-chain lengths (C12, C14, and C18) and found that all of them were able to produce Au nanomaterials (Supplementary Fig. 2). There has been some literature reporting that carboxylic groups act as reducing and stabilizing agents in the production of nanomaterials^18,19^. Here, the dense carboxylic groups on the membranes may also serve as reduction and stabilization agents for the gold nanosheets.

Moreover, when the mole ratio of C_Au_^3+^/C_DSA_ changed, the reaction mixtures showed different colours, indicating the presence of different sizes of Au^0^ nanostructures (Fig. 1a). The corresponding UV−Vis−NIR absorption spectra show the LSPRs of these nanomaterials (Fig. 1b). The gold nanomaterials were developed by means of progressive nucleation and isotropic or anisotropic growth, where the aqueous Au^3+^ initially nucleated into small nanoparticles (transmission electron microscopy images in Supplementary Fig. 3). For example, when the C_Au_^3+^/C_DSA_ mole ratio was 0.2 × 10^−3^, the synthesized Au^0^ solution was colourless and there was no obvious LSPR peak (Fig. 1b), indicating that the particle dimensions were <2 nm^20^. Upon increasing the HAuCl_4_ concentration, some particles experienced anisotropic growth into 2D nanosheets with a broad characteristic surface plasmon band at 900 nm belonging to in-plane dipole resonance from the plate-like structure (Fig. 1b and Supplementary Fig. 3)^21^. However, not all the isotropic particles formed in the initial states were fully converted into 2D nanosheets. There were both nanosheets and nanoparticles present in the field of view (Supplementary Fig. 3).

### Self-assembly at the liquid−liquid interface

After synthesis, we directly added water-insoluble organic solvent (ethyl acetate) to the prepared Au^0^ aqueous solution (C_Au_^3+^/C_DSA_: 5.7 × 10^−3^). Interestingly, the gold nanosheets together with some nanoparticles immediately self-assembled into a thin layer at the liquid (water)−liquid (ethyl acetate) interface (Fig. 1c–e). Other organic solvents, such as acetone, hexane, toluene, and dichloromethane, were also tested. Among them, only dichloromethane was able to trigger the self-assembly of the nanomaterials in the interface. It is noted that these nanosheet aggregates always returned to the liquid−liquid interface, even after violent shaking. However, for solution only with small particles (<30 nm), this phenomenon did not occur upon the addition of ethyl acetate (Supplementary Fig. 4, Supplementary Movie 2). Even upon decreasing the water volume, the nanomaterial aggregates remained at the interface to form spherical shapes until the solvents were removed (Fig. 1f–h). In this process, most of the DSA molecules were extracted out of the water phase into the organic solvent owing to their long water-insoluble alkyl chains. The liquid−liquid interface provides a mobile 2D platform for the self-assembly of the gold nanostructures. The driving force likely arises from the minimization of free energy, which was caused by thermal fluctuation and interfacial tension lowered by the DSA surfactants^22^.

**Fig. 2:**
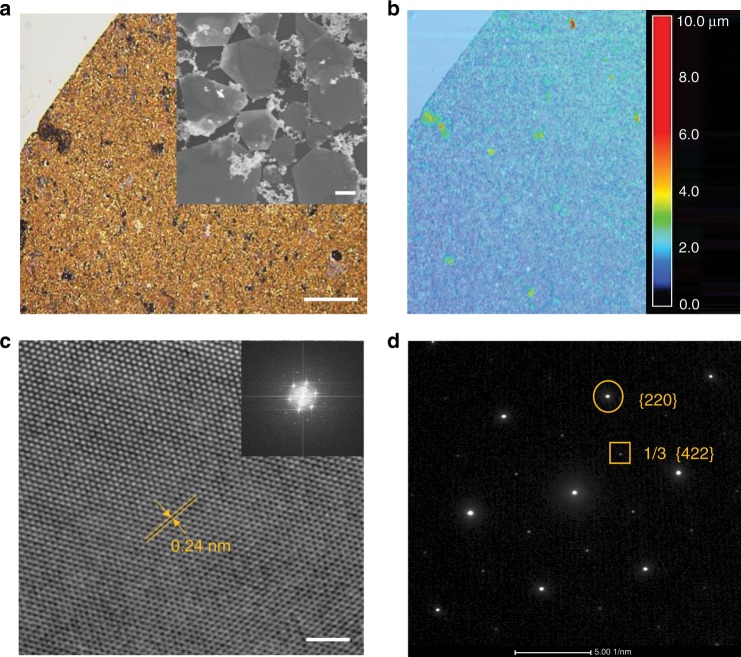
Structural characterizations of nanosheets directly transferred from the interface. **a**, **b** The as-prepared gold nanostructure assemblies at the interface were transferred onto a silicon wafer by pipette and subsequently detected under 3D laser scanning microscopy. The reflection microscope image (**a**) and 3D height map (**b**) of the gold nanostructure assemblies showed homogenous colour, indicating a relatively flat surface. The inserted SEM image in (**a**) showed that the gold nanostructure assemblies were mainly composed of nanosheets. Scale bar: 100 μm; inset: 1.0 μm. **c** A high-resolution transmission electron microscopy (TEM) image and (inset) corresponding fast Fourier transform diffraction pattern of a typical gold nanosheet. Scale bar: 2.0 nm. **d** Selected-area electron diffraction pattern of a gold nanosheet. The bright spot patterns were assigned to {220} (circled spot) and 1/3 {422} (boxed spot) Bragg reflections.

By using this interface method, the nanostructure assemblies were able to be easily transferred with a pipette onto various substrates (e.g. silicon wafer) to form a flat thin film. As detected by 3D laser scanning microscope, the average thickness of the assembled film was approximately 300 nm (Fig. 2a, b). Further characterizations with scanning electron microscopy (SEM, inset in Fig. 2a) and atomic force microscopy (AFM, Supplementary Fig. 5) showed that the films were mainly composed of nanosheets where the shape anisotropy was characterized by a ratio (*a*/*b*) of plate diameter *a* (several μm) to thickness *b* (~10 nm) as large as ~300. The nanosheet size distribution was shown in Supplementary Fig. 6. Furthermore, some nanoparticles were present in the field of view, as all the samples were analysed in their as-prepared state without any washing or separation methods (e.g. centrifuging). The shape anisotropy was mainly due to the DSA membranes acting as soft templating, which confined the growth of the gold crystals in a preferred direction on the bilayer membrane surface. In contrast, almost no nanosheet structures were formed, aside from nanoparticles, when a control experiment was performed at room temperature (Supplementary Fig. 7). This is because the DSA bilayer structure cannot form in water at a temperature below the Krafft point (~53 °C for C12-DSA). The Krafft point increases from 53 to 69 °C when the hydrocarbon chain length of DSA increases from 12 to 18. While if decreasing the hydrocarbon chain length to 4, no nanosheets but only nanoparticles can be synthesized using the same experimental condition (Supplementary Fig. 8). This is because DSA compounds with a short alkyl chain cannot form bilayer structure that is necessary for the anisotropic growth of gold nanosheets.

A high-resolution transmission electron microscopy (TEM) image was taken of a typical gold nanosheet, showing a lattice spacing of 2.4 Å (Fig. 2c). A selected area electron diffraction (SAED) pattern was obtained by focusing the electron beam on a typical nanosheet lying on the TEM grid. The hexagonal symmetry diffraction spot patterns were assigned to {220} and 1/3 {422} Bragg reflections corresponding to lattice spacings of 1.44 and 2.50 Å, respectively (Fig. 2d). The spot array characteristics indicate that the Au nanoplatelet is a single crystal with a preferential growth direction along the {111} plane. The presence of 1/3 {422} reflections are thought to be forbidden for a perfect face-centred-cubic (fcc) single-crystalline structure with an atomically flat surface^23^.

### {111}-oriented structure

**Fig. 3:**
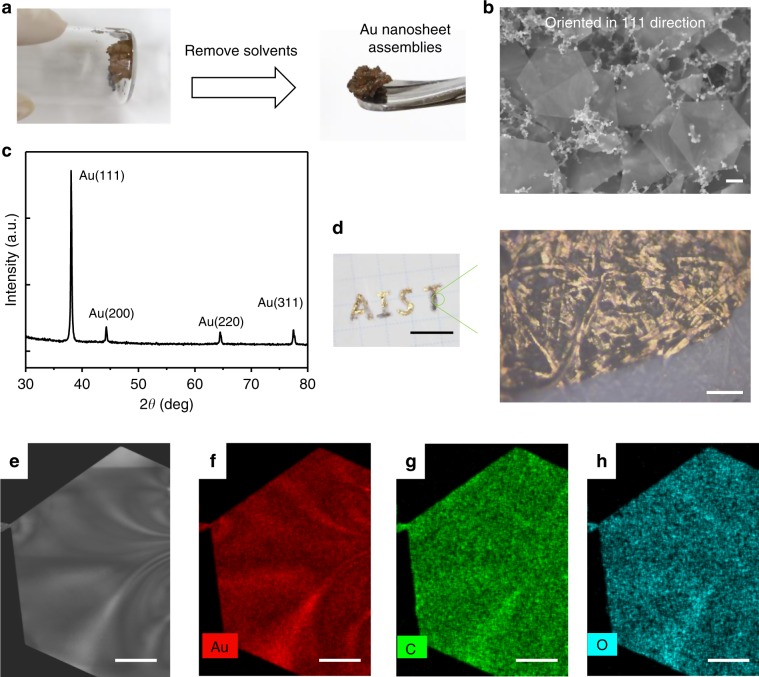
Morphological, structural and compositional characterizations. **a** The gold nanosheet aggregates were collected after removing the solvents by pipette. **b** Scanning electron microscopy image of the collected microscale (~5 μm) nanosheet aggregates. The bottom nanosheet can be seen from the top sheet, indicating its optical transparency. Scale bar: 1.0 μm. **c** X-ray diffraction pattern of the gold nanosheets. **d** The nanosheets were used as a paste to write characters on paper and observed under a reflection microscope. Scale bars: 1.0 cm (left); 100 μm (right). **e**−**h** Energy-dispersive X-ray spectroscopy elemental maps for Au (red), C (light green), and O (light blue) obtained from a gold nanosheet. Scale bars: 300 nm.

We collected the self-assembled nanostructure by removing the solvents and further observed them using SEM (Fig. 3a, b). The nanosheet aggregates were predominantly oriented parallel to each other, whereas the nanosheets prepared without the self-assembly strategy were randomly packed (Supplementary Fig. 9). The crystalline structures of the gold assemblies were also investigated by X-ray diffraction (XRD). As shown in Fig. 3c, the diffraction peaks were assigned to the Au {111} (38.1°), Au {200} (44.3°), Au {220} (64.6°), and Au {311} (77.5°) planes, corresponding to a typical fcc crystal structure. It is noted that Au {111} showed a strong peak intensity compared with those of the Au {200}, Au {220}, and Au {311} planes, suggesting that the synthesized Au nanosheets were dominated by {111} planes.

Furthermore, the collected nanosheet aggregates can be used as a paste to write characters on a flexible substrate. As shown in Fig. 3d, “AIST” was written on paper by a capillary tube. The reflection microscope image showed that the nanosheets were tightly integrated with the fibres of the paper.

When DSA is extracted by the upper ethyl acetate organic layer, some DSA may remain adsorbed on the nanosheet surfaces. To characterize this, we detected them using energy-dispersive X-ray spectrometry (EDX). As shown in Fig. 3e–h and Supplementary Fig. 10, the surfaces of the Au nanosheets were covered by carbon and oxygen elements from the DSA molecules. The estimated atom weight percentages from the EDX mapping spectrum are Au:(C and O) = 97.3%:2.7% (Supplementary Fig. 11).

### Mechanical properties

**Fig. 4:**
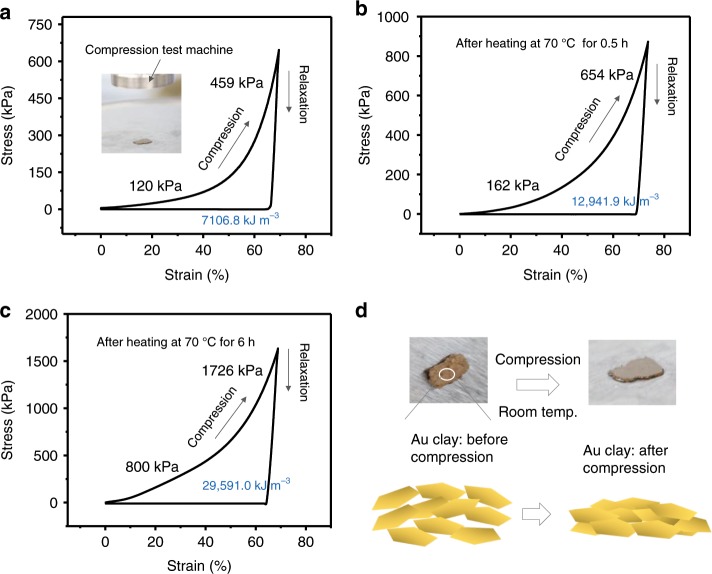
Plastic property of the soft gold metals from nanosheet assemblies. **a**–**c** Compressive stress−strain curves of gold nanosheet assemblies at room temperature (**a**) without annealing, and after annealing at a mild temperature of 70 °C for **b** 0.5 h and **c** 6 h. The hysteresis between compression and relaxation curves indicates plastic deformation. The calculated stiffness values at a strain of 10 and 60% are shown in the graphs. When thermal annealing is applied, energy dissipation (area between the loading and unloading curves) increases from 0.71 × 10^4^ to 2.96 × 10^4^ kJ m^−3^. **d** Images and schematic illustration of nanosheet assemblies under compressive stress.

A compression test was used to evaluate the plasticity of the Au nanosheet assemblies. As shown in Fig. 4a–d, the nanosheet aggregates show typical plastic deformation in that they deform during compression/loading and do not return to their original shape after the load is removed (relaxation). The area between the loading and unloading curves is the hysteresis area, which indicates energy dissipation during compression. When a gentle annealing (70 °C) was applied, a higher energy dissipation (2.96 × 10^4^ kJ m^−3^) was observed in the materials during dehydration, which was mainly due to increased interfacial friction between the nanosheets.

During the initial 0–40% compression strain (*Ɛ*), the slope of the stress−strain curves (stiffness, *σ*/*Ɛ*) gradually increased. In contrast, upon a large deformation (60–70%), the slope became sharp (e.g. high stiffness value up to 459 kPa in Fig. 4a), indicating a hardening process of the materials due to a decrease in layer distance (densification). This was also confirmed by the cross-sectional SEM images of the sample after applying stress (strain ~60%). The distance between Au nanosheets (porous) decreased to hundreds of nanometres (Supplementary Fig. 12). If the strain were further increased, the distance would be further decreased. The catalytic activity of this porous structure (specific surface area ~1.49 m^2^ g^−1^) was also evaluated (Supplementary Fig. 13). The catalytic efficiency was not competitive with other noble metal nanoparticles, and this was probably because the active catalytic sites were blocked by the DSA molecules on the surface of nanostructures.

The stiffness of the nanostructure without annealing was approximately several hundred kPa and the materials after gentle annealing showed stiffness values up to several thousands of kPa at a strain of ~60% (Fig. 4a, c). Although annealing hardened the materials, their stiffness was much lower than the room-temperature value for bulk gold of *E* = (79 ± 1) × 10^6^ kPa^24^. The stiffness values indicate that these nanosheets are about six orders of magnitude softer than bulk gold. The microscale, thin nature of the nanosheets covered by DSA (as an organic binder) is beneficial to the final softness of these hybrid metal materials.

### Macroscale metallic architectures build from nanostructures

**Fig. 5:**
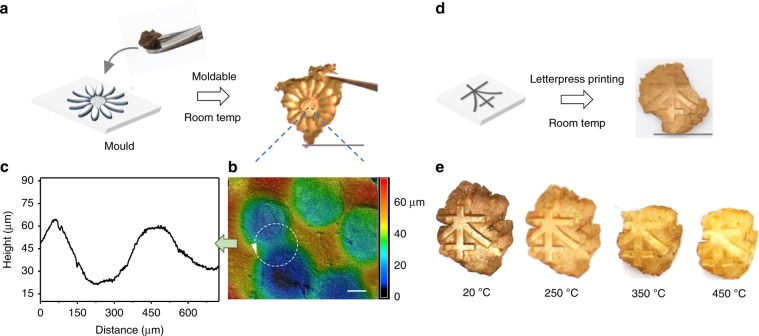
Mouldable gold metal clay. **a** Demonstration of a free-standing moulded shape from the gold nanosheet aggregates at room temperature. **b** Surface 3D height maps of the structure in the circled area from (**a**). **c** The corresponding surface profiles along the white dashed lines in (**b**). **d** Demonstration of macroscale moulded characters from gold clay at room temperature and **e** annealing of the deformed shape under high temperatures. Scale bars: **a** 1.0 cm; **b** 100 μm; **d** 1.0 cm.

The materials are highly mouldable despite being composed of 97% metal by weight (Supplementary Fig. 11). Therefore, we call these soft hybrid materials as gold clay. As a demonstration, we produced a flower-shaped metal structure using the gold clay (Fig. 5a). The gold clays were covered on the surface of a mould (flower pattern from a coin) and then pressed by hand for 2–5 s at room temperature. The obtained gold clay structures could be gently peeled off from the mould to give a macroscale free-standing patterned metal without any additional thermal annealing (Fig. 5a).

To the best of our knowledge, to produce a macroscale free-standing metal film at room temperature from nanostructure building blocks remains a challenge. Most reported nanostructured metal films must be transferred or deposited to a substrate, such as polyurethane film^6^ or other solid-based materials^25–27^. Although 3D printing can produce metallic architectures from nanoparticle ink, a continuous thermal annealing using focused laser irradiation is necessary to firm the free-standing architectures^28,29^. In this system, the surface attractive force (e.g. hydrogen bonding from carboxylate groups of DSA) may increase the adhesion of the nanostructures. Furthermore, the self-assembly strategy for producing oriented structure and interlaced microscale 2D shape is beneficial to produce these deformable and free-standing hybrid metals without any thermal annealing. This is also inspired by existing natural clays, which also have oriented packing structures and microscale sheet shapes (Supplementary Fig. 14).

As shown in Fig. 5b–c, the structural details of the flower-shaped mould were clearly achieved in the gold clay, even for a height difference of only tens of micrometres, indicating its ability to produce exquisite patterns. These soft gold clays were able to be moulded into arbitrary shapes or patterned masters, e.g. in letterpress printing. As shown in Fig. 5d, the characters were clearly visible on the gold metals.

To investigate high-temperature dehydration effects, the patterned metallic architectures (prepared at room temperature) were further hardened under annealing. As shown in Fig. 5e, when the temperature was below 250 °C, there was no significant difference in the patterns or the overall dimensions of the metal clays. However, the colour became more golden and hardness increased significantly, owing to the absorbed DSA molecules (~3wt.%) gradually decomposing at temperatures above 200 °C, as indicated by thermogravimetric spectrum (Supplementary Fig. 15). As hardening continued at temperature above 350 °C, the overall dimensions of the metals decreased ~10%, especially in the thickness. The anisotropic shrinking of the metal clay caused the sample to curve slightly to one side when the temperature was above 450 °C. But it is worth noting that it still maintains the structural details as the character was still visible, and the hardness was close to the bulk gold.

### Stress-dependent conductivity and morphology change

**Fig. 6:**
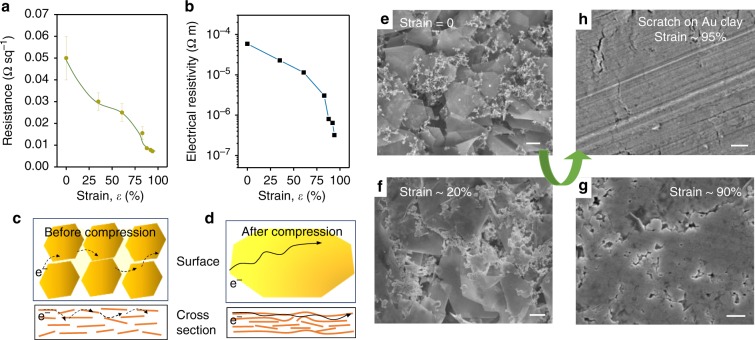
Stress/strain-dependent conductivity and morphology change during compression. **a**, **b** Stress/strain-dependent electrical conductivity of the gold nanosheet aggregates. Note that no thermal annealing was applied during measurements. Error bars are defined as s.d. **c**, **d** Schematic illustrations of the surface and cross-sectional changes of the gold nanosheet aggregates before and after mechanical compression. **e**–**h** Scanning electron microscopy images of surface morphologies with increasing stress/strain at room temperature. Scale bars: 1.0 μm.

In addition to plasticity, mouldability, and stress/strain or temperature-induced hardening, the gold clays are highly conductive (Supplementary Movie 3). The detailed resistance and electrical resistivity (*ρ*) values are shown in Fig. 6a, b. The gold clay was deformed into a square-shaped sample, and changes in strain were precisely controlled by a tensile machine. Before and after every pressing (without any thermal treatment), the surface resistance values were measured by the four-probe method with a low resistivity metre (Loresta-AX MCP-T370 Mitsubishi Electric) at room temperature. The electrical resistance value (*ρ*) decreased from ~5 × 10^−5^ Ω m (as-prepared state, thickness: 1.17 mm) to 3.2 × 10^−7^ Ω m (after pressing to ~0.09 mm) with increasing compressive strain (Fig. 6a, b). Although the electrical resistance value (*ρ* = 3.2 × 10^−7^ Ω m) of the pressed gold clay is one order of magnitude higher than that of bulk gold (*ρ*_bulk_ *=* 2.44 × 10^−8^ Ω m), it is close to the value of a sputtered gold film or even lower than a typical thermally evaporated Au thin film^30,31^. Furthermore, compared with gold clay from nanoparticles that exhibit electric properties close to semimetals^6^, this gold clay composed of microscale nanosheets that exhibited a highly improved conductivity. This is probably because conductivity and plasticity have a trade-off relationship. The gold clay derived from nanoparticles requires more organic binders to maintain its plasticity than microscale nanosheets that have a strong surface interlace adhesion.

The increase in conductivity upon mechanical compression at room temperature is mainly due to the stress-induced surface and cross-section morphology changes (Fig. 6c, d). The gold clay before compression has a dark brown colour with loose nanosheet aggregation, whereas it becomes a compact and fused surface with a shining golden colour after compression (Supplementary Fig. 16). There have been some interesting studies that show new nanostructures, such as gold or CdSe nanowires can be synthesized by compression of their small nanoparticles^32–34^. In this work, we found that large area of compact and fused morphology changes appears in nanosheet assemblies upon mechanical compression. The gold clay composed of thin nanosheet assemblies gradually forms a smooth surface with less grain-boundary sliding and smaller gaps among the nanosheets upon increasing stress/strain (Fig. 6e–h). This likely accelerates electron transport and thus increases the electrical conductivity of the material. Additionally, the presence of nanoparticles also helps to improve the connectivity between the nanosheets, thus affecting electrical properties of the self-assembled material upon mechanical compression.

### Potential applications

The gold clay mainly composed of 2D microscale nanosheets is expected to open various application opportunities. Firstly, the gold clay (as a paste) is able to be produced into designed free-standing metallic architectures with microscale resolution at different sizes (Supplementary Fig. 17, Supplementary Movie 4). For example, the produced stripe pattern has a width of 100 μm and height of 50 μm. It is important to note that the patterned metallic architectures become firm upon mechanical stress (without any thermal annealing) probably because of the surface morphology change of oriented nanostructures under compression and interlaced adhesion of the nanosheets. The ability to be patterned into high-resolution, free-standing complex metallic structures may open up new avenues for creating conductive electronics and devices^29^. The second interesting point is that these materials show stress/strain-dependent electrical conductivity, which has potential application in stress detecting. The third application of these materials is for electric wiring on flexible substrates (Fig. 3d). Although gold is an expensive material, they are chemically stable. For example, the gold clay can be used as conductive sealing paste on electronics in an acidic or alkaline environment. In addition, there are other interesting applications, such as using these gold clay for making thin gold foils (Supplementary Fig. 16).

The organic binders on the surface of the nanostructures are advantageous for achieving the aforementioned interesting applications, such as flexible moulding. However, this feature is disadvantageous for the application as catalysis because the active catalytic sites will be blocked by the organic binders.

## Discussion

In summary, we demonstrated a deformable gold clay fabricated by self-assembly of microscale-oriented nanosheets. The single-crystalline gold nanosheets were synthesized between DSA bilayer membrane structures in water. The nanosheets can automatically grow up to a microscale size on the bilayer membrane surface. The gold clay mainly composed of {111}-oriented nanosheets from self-assembly at the liquid−liquid interface demonstrates high plasticity and softness. Interestingly, they can be easily deformed into arbitrary shapes or patterns at room temperature. The patterned metal did not lose its structural details, even after hardening at a high temperature. Furthermore, the pressed gold clays are highly conductive, with a conductivity value comparable to that of bulk gold. This study may attract immense interest in fundamental research because the developed experimental route shows high level of simplicity concerning the chemical synthesis of 2D nanostructures as well as for the self-assembly process on fabrication of metal clays. Moreover, the ability of using these materials to produce free-standing metallic architectures with microscale resolution at different sizes may have technological significance for various applications, including flexible electronics, devices, sensors, decoration, and metal foil fabrications with reduced cost and complexity.

## Methods

### Controlled growth of gold nanosheets

The gold nanosheets were synthesized in a DSA aqueous solution with bilayer membrane structures. DSA compounds with different alkyl chain lengths were synthesized by hydrolysis of respective succinic anhydrides (see Supplementary Note 1). The pure DSA compounds (white powder) were dissolved in water to 1–1.2 wt%. The aqueous solutions were then kept in a glass bottle in a water bath at a temperature above their respective Krafft points (e.g. C12-DSA, *T* = 53 °C, pH ≈ 3.3) for tens of minutes to achieve a homogeneous solution (with a stable and uniform phase of bilayer structures). Then, a small amount (500 μl) of HAuCl_4_ solution (2 × 10^−3^ M) is added into the DSA aqueous solutions (5 ml). The bottle is gently shaken to mix the solution in a mild way. After mixing the solution, the bottle is immediately kept in water bath (53 °C) without any stirring. After 15 min, the red-wine colour of the solution appears, indicating the nucleation and growth of the gold nanomaterials. The plate diameters increase with the reaction time. When the size grows into a large microscale size (e.g., ~5 μm, 1.5 h), the nanoplates gradually settle down by themselves at the bottom of the bottle.

### Synthesis of gold clay

Ethyl acetate was added to the above solution and then the nanosheets were automatically self-assembled at the liquid−liquid interface. The gold clay was prepared by drawing the solvents (ethyl acetate and water) using a pipette from the bottom. During drawing the solvents, they kept in the water/ethyl acetate interface, and assembled together when all of the solvents were removed. The assembled nanosheets (gold clay) were used for moulding into different metallic architectures immediately or after drying at room temperature for several minutes. The solvent content in the gold clay (as-prepared state) was about 40 wt%.

### Characterizations

The UV–Vis absorption spectrum was collected using a JASCO V-670 spectrophotometer (JASCO, Japan). The 3D laser scanning microscopy images were taken using a Keyence VK-X100 laser microscope (KEYENCE, Japan) with a laser wavelength of 658 nm. Scanning electron microscopy images were obtained on a field emission scanning electron microscope JSM-6340F (JEOL, Japan) at an operating voltage of 10 kV. Atomic force microscopy images were taken using a nano search microscope SFT-3500 (SHIMAZU, Japan). Transmission electron microscopy was measured on a Tecnai Osiris (USA), which was equipped with an energy-dispersive spectroscopy detector. X-ray diffraction data were collected on a SmartLab Rigaku-X-ray analytical machine (USA) with Cu Kα (*λ* = 1.5418 Å). The compressive stress−strain curves were acquired using a tensile machine (Tensilon EZ-LX, SHIMAZU, Japan). The specific surface area was measured via the Brunauer–Emmett–Teller (BET) method using a BELSORP-mini (BEL Japan, Inc.). Thermogravimetric measurements in dynamic conditions were carried out using SII Nanotechnology DSC6100. Other detailed information on the characterizations is provided in Supplementary Note 2.

## Supplementary information

Supplementary Information

Description of Additional Supplementary Files

Supplementary Movie 1

Supplementary Movie 2

Supplementary Movie 3

Supplementary Movie 4

## Data Availability

The authors declare that all data supporting the findings of this study are available within the paper and its supplementary information files or from the corresponding authors upon reasonable request.
