# Supplementary material for: Gold clay from self-assembly of 2D microscale nanosheets
Source: Nat Commun. 2020 Jan 29;11:568. doi: 10.1038/s41467-019-14260-5 (PMC6989663; doi:10.1038/s41467-019-14260-5)
Supplement: Supplementary file 1 — Supplementary Information [file 41467_2019_14260_MOESM1_ESM.pdf]

Supplementary Information

Soft gold clay from self-assembly of 2D microscale nanosheets

Yue et al.

Supplementary Note 1

Synthesis and NMR data of DSA compounds. DSA compounds with different alkyl-chain length (C12, C14, C18) were synthesized by hydrolysis of their succinic anhydride in NaOH water solution (6M) at a temperature of 90-100°C with strong stirring. The reaction was stopped by adding large amount of water and cooling to room temperature. Then, pH values of the mixture were adjusted to about 2 by adding HCl solution (2M) drop by drop. The compounds were extracted by ethyl acetate and washed with water for several times. The pure compounds were recrystallized in acetone and characterized by nuclear magnetic resonance ^{1}H NMR and ^{13}C NMR on a Bruker Advance III NMR spectrometer at a resonance frequency of 500 MHz. The residual protonated solvent signals were used as internal references for ^{1}H NMR spectra (^{1}H NMR: δ ($CDCl_3$). 7.26 ppm and ^{13}C NMR: δ ($CDCl_3$). 77.16 ppm)

Compound C12: ^{1}H NMR (500 MHz, $CDCl_3$) δ 5.48-5.54 (m, 1H), 5.28-5.34 (m, 1H), 2.85-2.89 (m, 1H), 2.64-2.72 (m, 1H), 2.50-2.54 (m, 1H), 2.42-2.47 (m, 1H), 2.19-2.25 (m, 1H), 1.96-2.00 (m, 2H), 1.25-1.32 (m, 14H), 0.88 (t, 3H); ^{13}C NMR (125 MHz, $CDCl_3$) δ 180.9, 178.7, 134.9, 125.1, 41.3, 34.8, 34.6, 32.5, 31.9, 29.6, 29.5, 29.3, 29.1, 29.0, 27.32, 22.7, 14.1.

Compound C14: ^{1}H NMR (500 MHz, $CDCl_3$) δ 5.48-5.54 (m, 1H), 5.28-5.34 (m, 1H), 2.85-2.89 (m, 1H), 2.64-2.69 (m, 1H), 2.50-2.55 (m, 1H), 2.41-2.47 (m, 1H), 2.19-2.25 (m, 1H), 1.96-1.99 (m, 2H), 1.25-1.32 (m, 20H), 0.87 (t, 3H); ^{13}C NMR (125 MHz, $CDCl_3$) δ 180.9, 178.7, 134.9, 125.0, 41. 3, 34.8, 34.6, 32.5, 31.9, 29.7, 29.7, 29.6, 29.5, 29.4, 29.3, 29.2, 27.3, 22.7, 14.1.

Compound C18: ^{1}H NMR (500 MHz, $CDCl_3$) δ 5.48-5.54 (m, 1H), 5.29-5.34 (m, 1H), 2.85-2.89 (m, 1H), 2.63-2.69 (m, 1H), 2.50-2.54 (m, 1H), 2.43-2.45 (m, 1H), 2.20-2.25 (m, 1H), 1.96-1.99 (m, 2H), 1.21-1.32 (m, 28H), 0.87 (t, 3H); ^{13}C NMR (125 MHz, $CDCl_3$) δ 180.7, 178.5, 134.9, 125.05, 41.3, 34.8, 34.5, 32.5, 31.9, 29.7, 29.6, 29.5, 29.3, 29.1, 22.7, 14.1.

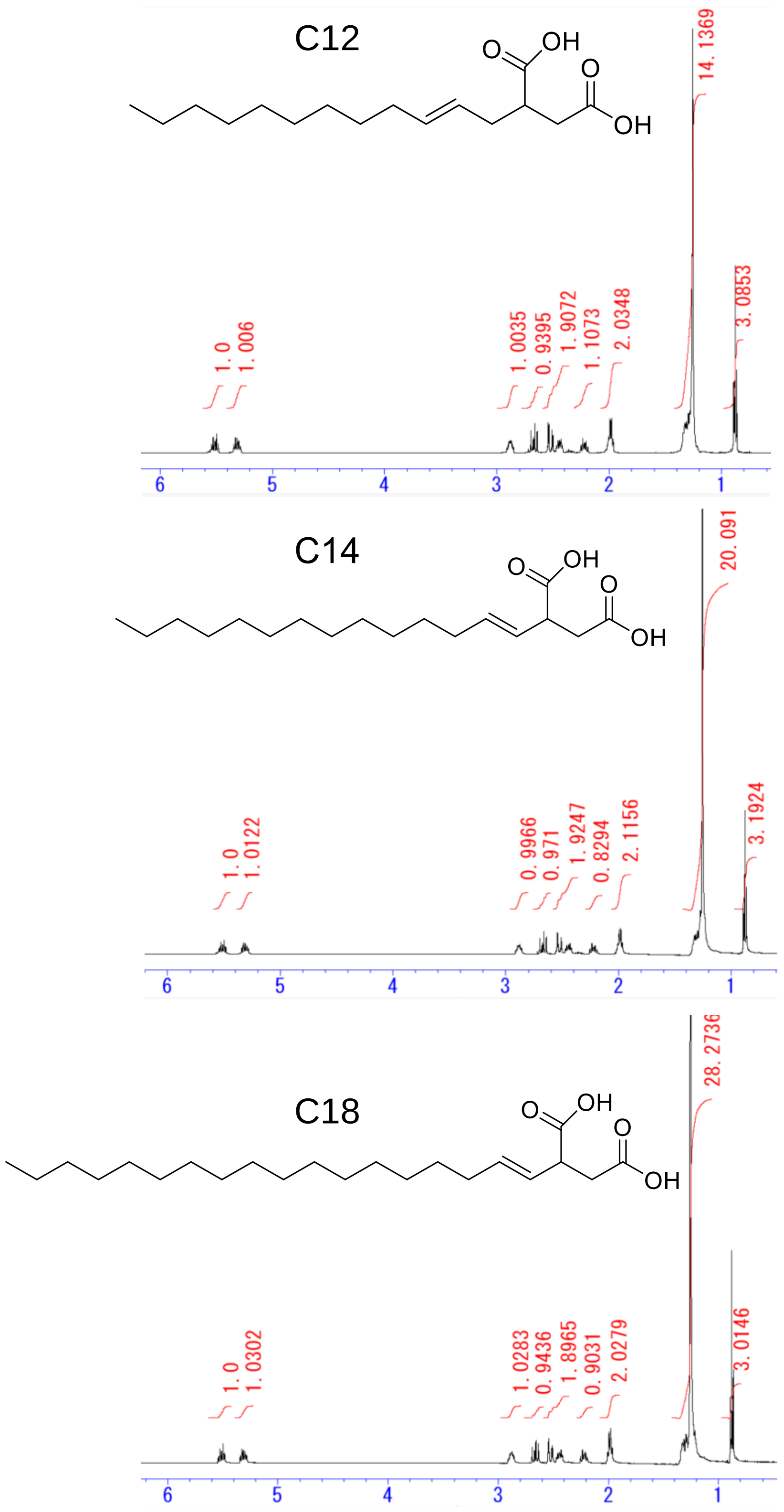

^{1}H NMR data and chemical structures of DSA compounds with different lengths of alkyl chain (C12, C14 and C18)

Supplementary Note 2

UV–Vis absorption spectra: The spectrum was collected using a JASCO V-670 spectrophotometer (JASCO). The measurements were made using a double-beam spectrophotometer with 1-cm quartz cuvettes and water was used as the blank.
3D laser scanning microscopy: The images were taken using a Keyence VK-X100 laser microscope with a laser wavelength of 658 nm.
Scanning electron microscopy: SEM images were observed with a field emission scanning electron microscope JSM-6340F (JEOL, Japan) at an operating voltage of 10 kV and current of 10 μA. For the image of the thin film (e.g., Fig. 2a), the nanosheets were directly transferred from the liquid-liquid interface to silicon wafer with a pipette. For images of the thick nanosheet assemblies, (e.g., Fig. 3b), the nanosheets were transferred to the silicon wafer after removing all the solvents.
Atomic force microscopy: Observation was made by a nano search microscope SFT-3500 (SHIMAZU, Japan).
Transmission electron microscopy: The samples were observed using a TEM (Tecnai Osiris, FEI) which was equipped with an energy-dispersive spectroscopy (EDX) detector, and operated in scanning transmission electron microscopy mode with a probe diameter of ~0.3 nm.
X-ray diffraction: X-ray diffraction data for the free-standing film were collected on a SmartLab Rigaku-X-ray analytical machine with Cu Kα (λ=1.5418 Å). The measurement was carried out using an X-ray generator with a voltage of 45 kV and a current of 120 mA. The scan runs at a step size of 0.01°2θ. The sample was directly placed on the detecting substrate. X-RD data were collected for the same polymer after UV irradiation at the same scanning conditions.
Compressive stress-strain testing: The compressive stress-strain curves were acquired using a commercial machine (Tensilon EZ-LX, SHIMAZU, Japan) with a load cell of 100N.
Gas adsorption measurements: Gas adsorption analysis is commonly used for surface area and porosity measurements. The surface area studies of the sample were carried out using a BELSORP-mini (BEL Japan, Inc.). The samples were first degassed at 100 °C for more than 3h to remove the contaminants such as water and other adsorbed gases. The sample was then subjected to analysis and the surface area was recorded by using Brunauer-Emmett-Teller method. The pore value of the sample was analyzed by Barrett–Joyner–Halenda (BJH) method. Nitrogen gas was used as adsorbent.
Thermogravimetry: Thermogravimetric measurements in dynamic conditions were carried out in the temperature range of 30–450 °C at constant heating rate 10 °C min^{-1} using SII Nanotechnology DSC6100.

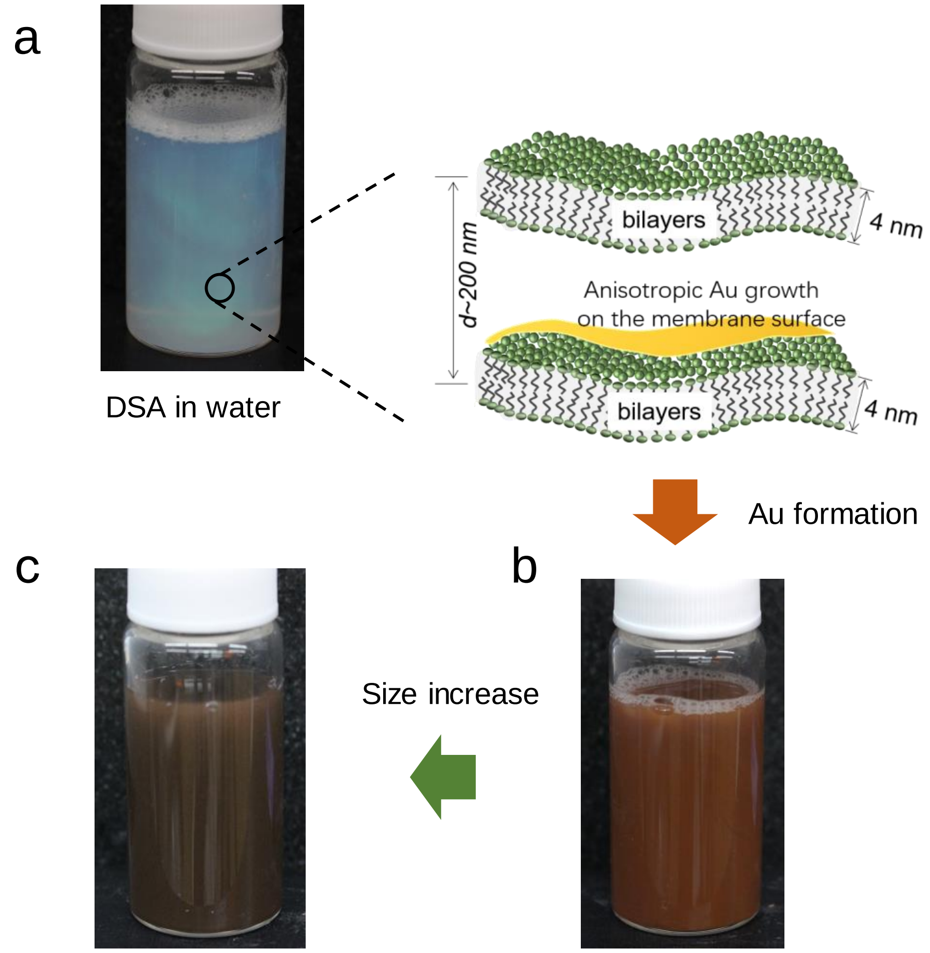

Supplementary Fig. 1 Scheme illustration and images of the controlled growth of gold nanosheet on the surface of DSA bilayer membranes. (a) The pure DSA aqueous solution (1–1.2wt%) shows iridescent structural color due to the visible light diffraction on the periodic bilayer structure with a layer distance about 100–200 nm, which can be estimated from Bragg's law $\lambda=2nd\sin\theta$ (where n is the average refractive index of water, d is the distance of the layer, and θ is the Bragg glancing angle). The thickness of the bilayer is about 4 nm for C12, 4.5nm for C14, and 5.6 nm for C18, which can be calculated from the chemical structure of the compounds from Chemical Draw professional 16.0. (b, c) When keeping the bottle (with a mixture of DSA, water and $HAuCl_4$) in the water bath (53 °C), the colour changed dramatically, indicating the size growth of the nanomaterials.

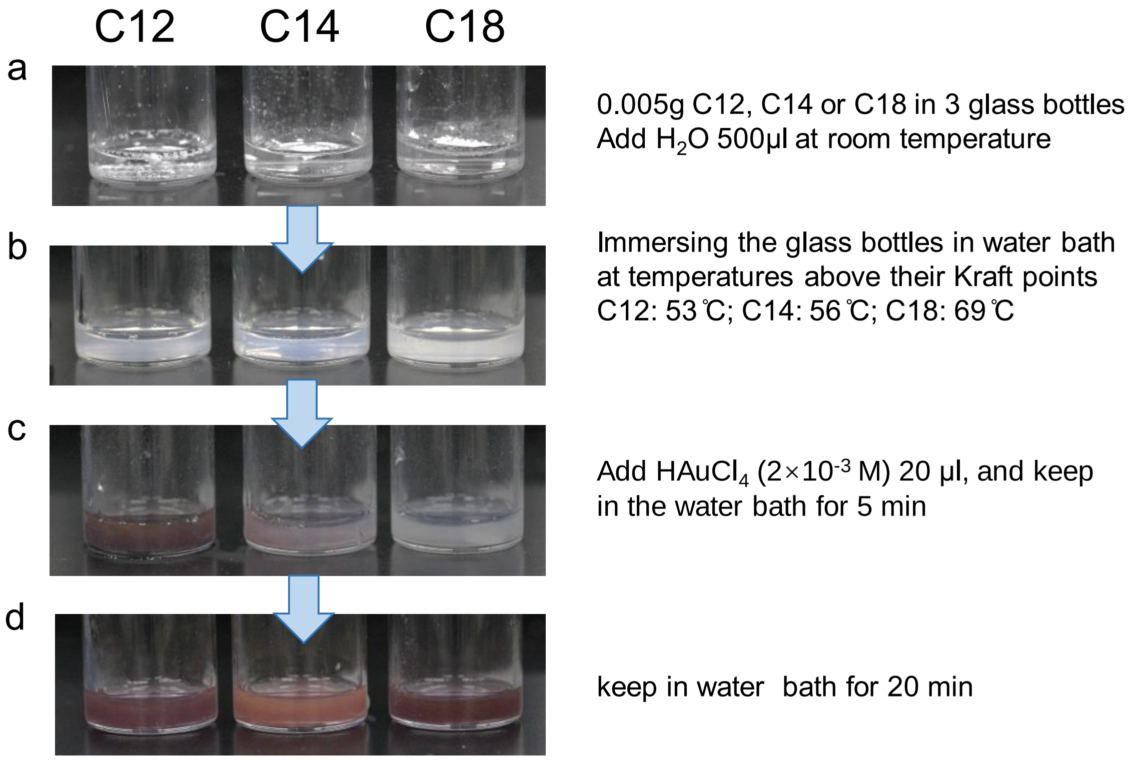

Supplementary Fig. 2 The DSA with different lengths of alkyl chain (C12, C14, and C18) in the synthesis of Au nanomaterials. (a) DSA compounds (0.005g) with different lengths of alkyl chain in water (500μl) at room temperature. (b) The samples were heated in the water baths at temperatures close to the Krafft temperatures of the compounds. (c, d) $HAuCl_4$ (2×10^{-3} M) was added to the solutions and kept the bottles in the water bath for 5 and 20 min.

Krafft point here is the minimum temperature at which the surfactants form bilayer structure in water. It depends on the nature of hydrophobic groups and ionic composition of the compounds.

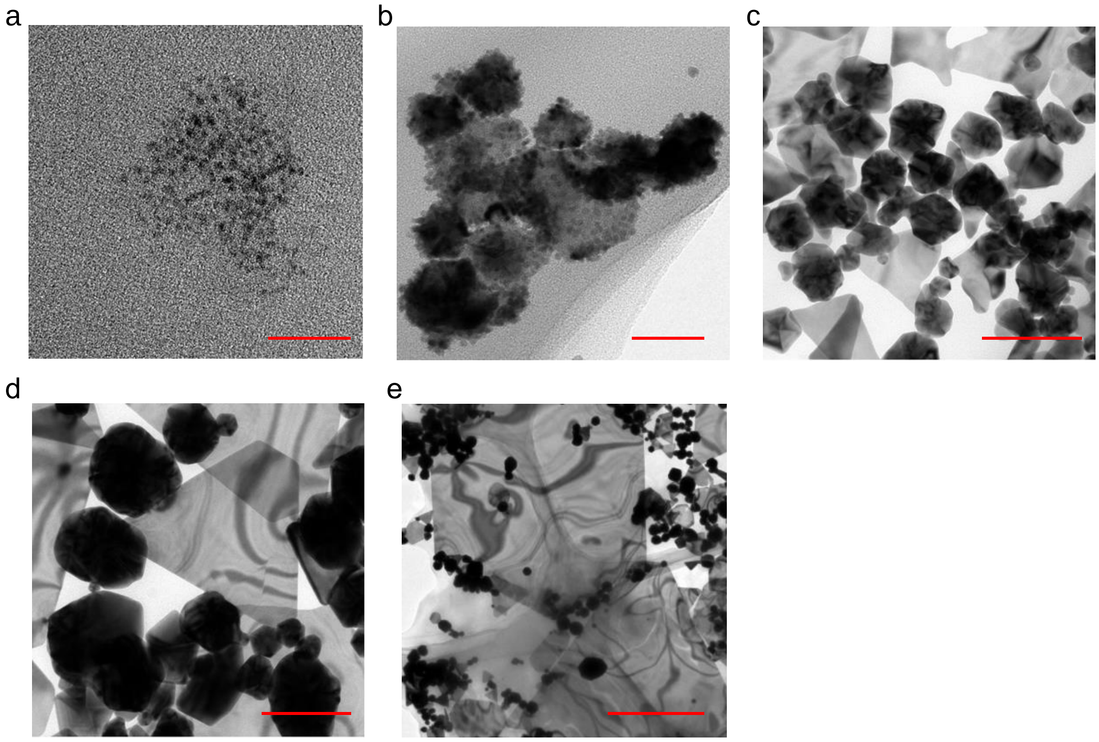

Supplementary Fig. 3 Transmission electron microscopy (TEM) images of the gold nanomaterials at different mole ratios of $C_{Au}{}^{3+}/C_{DSA}$. From left to right $C_{Au}{}^{3+}/C_{DSA}$ mole ratio: (a) 0.2×10^{-3}, (b) 0.6×10^{-3}, (c) 1.0×10^{-3}, (d) 2.0×10^{-3}, and (e) 4.0×10^{-3}. When the $C_{Au}{}^{3+}/C_{DSA}$ mole ratio was 0.2×10^{-3}, the synthesized Au^0 particle dimensions were <2nm. When the mole ratio of $C_{Au}{}^{3+}/C_{DSA}$ is $\geq1.0\times10^{-3}$, there are some nanosheets appears. Please note that not all the isotropic nanoparticles formed in the initial states are fully converted into 2D nanosheets. There are nanoparticles surround nanosheets. The size of the nanosheets increase significantly with the increasing in the ratio of $C_{Au}{}^{3+}/C_{DSA}$. Scale bars: (a) 20 nm, (b) 50 nm, (c) 100 nm, (d) 100 nm and (e) 1.0 µm.

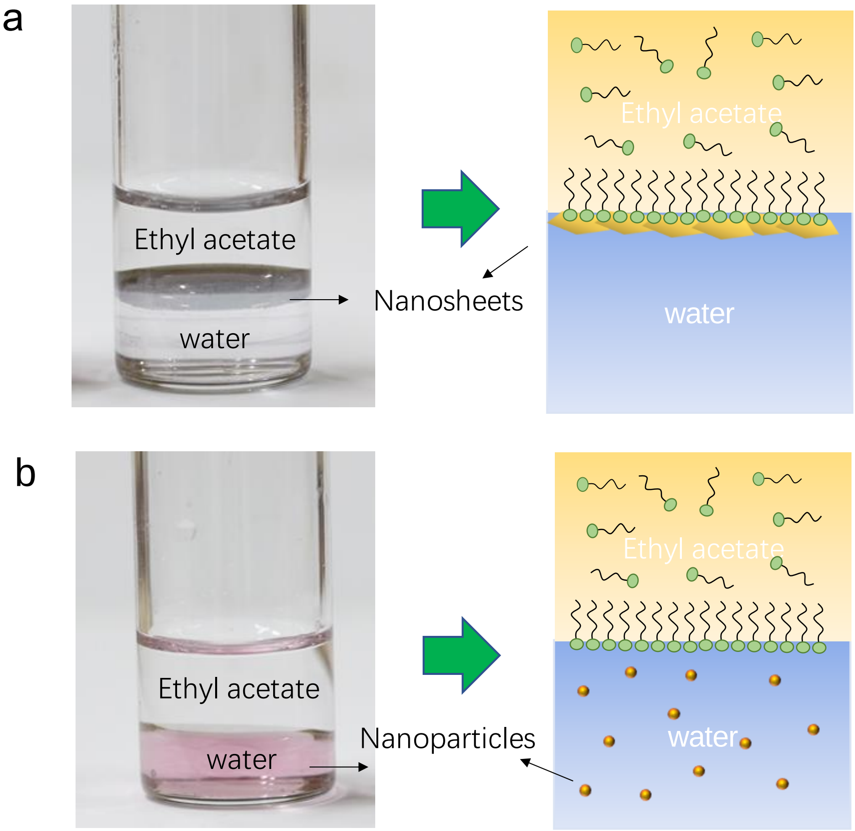

Supplementary Fig. 4 Self-assemble of Au nanosheets at the liquid-liquid interface. (a) Photographs and scheme illustration of the Au nanosheet aggregate in the interface of water/ethyl acetate. (b) Photographs and scheme illustration of Au nanoparticles (~25 nm) only existed in the bottom water layer after adding ethyl acetate. In both case, most of the DSA surfactants are extracted into the organic layer. Some DSA molecules are in the interface with their hydrophilic carboxylic groups in the water phase and hydrophobic alkyl chain in the organic phase.

Other organic solvents such as hexane, toluene, acetone, and dichloromethane were tested. Ethyl acetate and dichloromethane were found to be good solvents for inducing self-assembly of the gold nanosheets. Some effects, such as the solubility of DSA in organic solvents, the compatibility of organic solvents with water, and the size of the nanomaterials may affect the self-assembly.

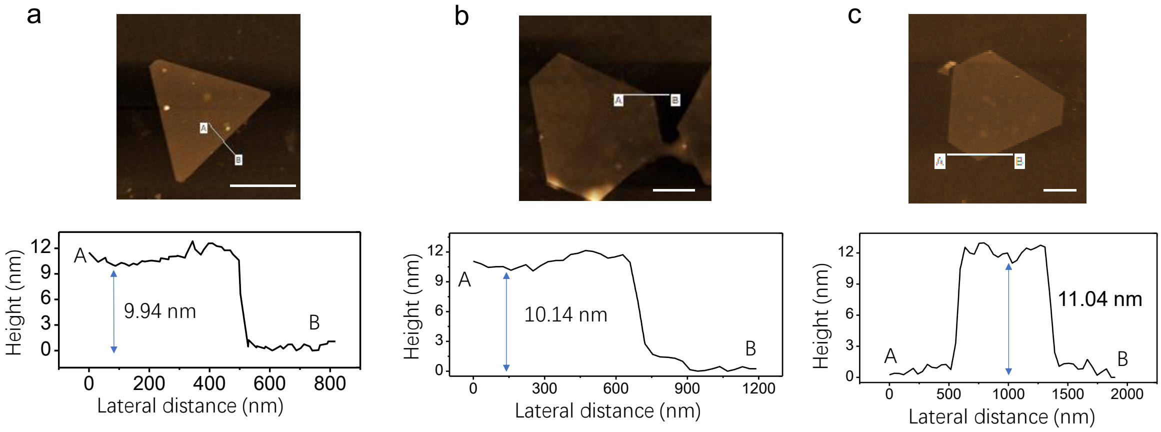

Supplementary Fig. 5 Atomic force microscope (AFM) images of gold nanosheets and a line profile of the area in the nanosheet indicated by the line in the AFM image. The shape anisotropy values, characterized by a ratio of plate diameter to thickness (a/b) are (a) 167, (b) 308, and (c) 322. Scale bars: 1.0 µm.

In this study, using DSA bilayer as template, it is easy to synthesis thin microscale nanosheets compared with other reported methods. It is probably because the growth speed along the diameter on the membrane surface is much bigger than the growth speed along the thickness direction. Typically, during the growth of gold nanosheet, the thickness increases with the plate diameter. For example, when the diameter of the gold nanosheet is 100-200 nm, the thickness is 10 nm;[1] when the diameter increases to 400 nm, the thickness increases to >20 nm;[1] in most cases, the thickness is > 20 nm when the diameter increases to micrometers.[2-6]

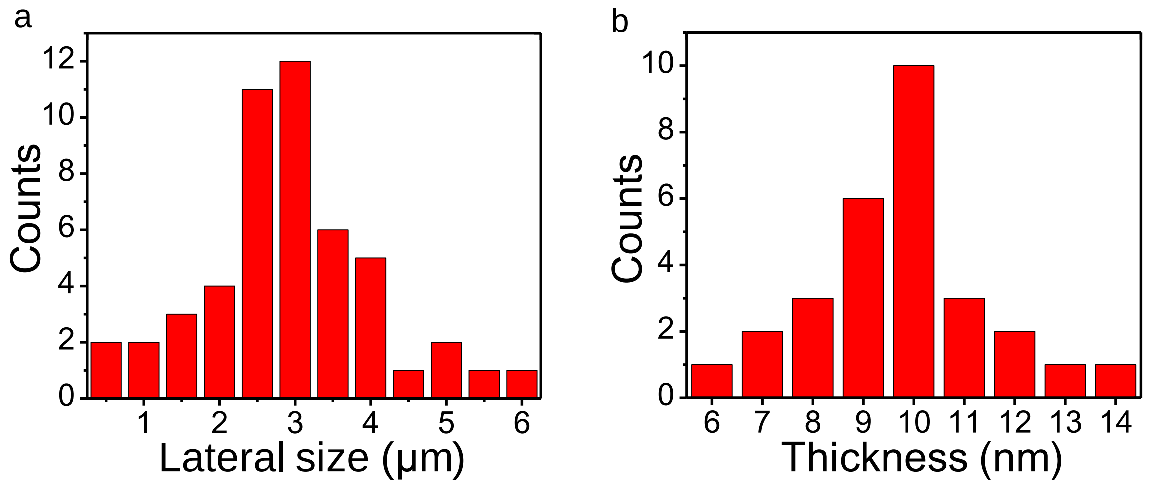

Supplementary Fig. 6 The histogram of particle size distribution of the gold nanosheets when the mole ratio $C_{Au}{}^{3+}/C_{DSA}$ is 5.7× 10^{-3}. (a) Lateral size. (b) Thickness.

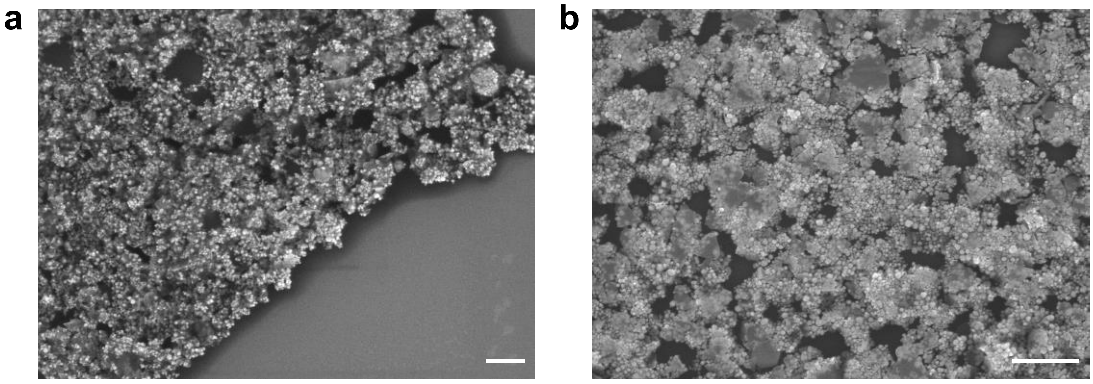

Supplementary Fig. 7 Scanning electron microscopy (SEM) images of the gold nanomaterials synthesized by the same method from DSA aqueous solution at room temperature.

In order to study the role of temperature on the growth of gold nanomaterials, a control experiment was performed. In this experiment, the reaction bottle was moved out of the water bath (53°C) to room temperature after DSA aqueous solution was prepared. Then we added $HAuCl_4$ aqueous solution and kept the bottle in room temperature overnight. Other conditions were the same as in the main text. As shown in Supplementary Fig. 7a-b, small amount of nanosheets were produced except nanoparticles with size about 100 nm. This experiment indicates that bilayer structure is important as a template for the 2D controlled growth of nanosheets, because DSA bilayers cannot exist in water at room temperature. This experiment also indicates that the DSA compound can reduce Au^{3+} to Au^0 even at room temperature. The reducing speed is slow (observing colour after keeping for 2-3h) compared with that at 53°C (15min). In addition, without bilayer structure, the reduced gold are mainly nanoparticles rather than nanosheets. Scale bars: 1.0 µm.

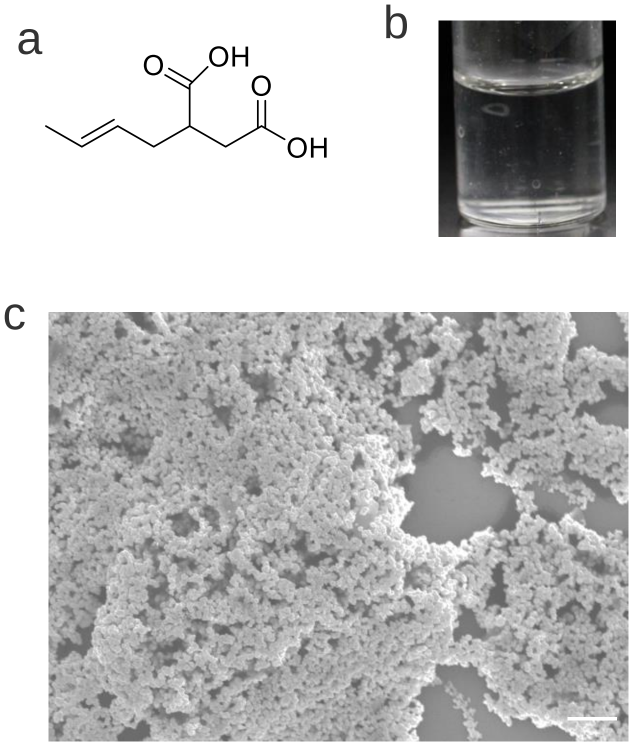

Supplementary Fig. 8 SEM images of the gold nanomaterials synthesized by using a C4-DSA compound in water. No nanosheets but nanoparticles were synthesized by using C4-DSA in water, indicating that the DSA bilayer structure is important for the anisotropic growth of nanosheets. C4-DSA compound (with a short alkyl-chain) cannot form bilayer structure in water and thus the solution shows transparent. (a) Chemical structure of C4-DSA. (b) 1.1 wt% C4-DSA in water. (c) The synthesized gold nanomaterials by using C4-DSA. Scale bar: 1.0 μm.

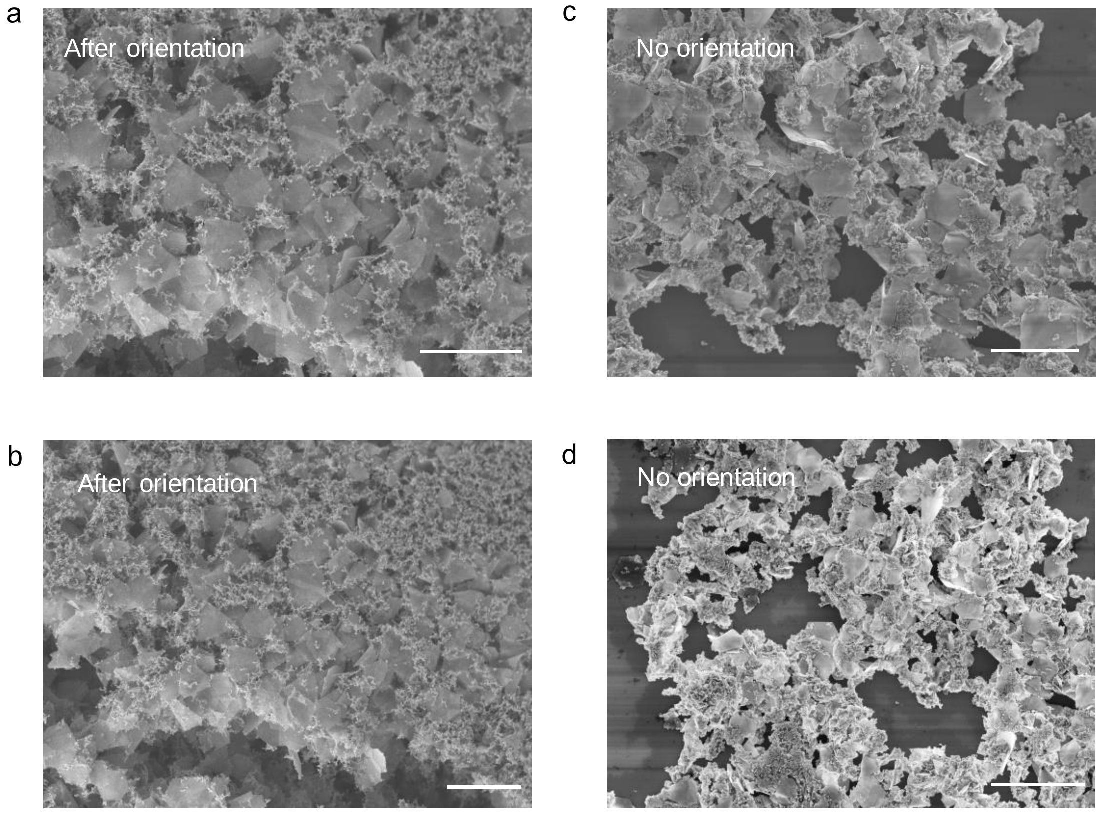

Supplementary Fig. 9 SEM images of the gold nanosheets with and without interface induced self-assembly. (a, b) The nanosheets were prepared and aggregated by self-assemble strategy using the liquid-liquid interface (via adding ethyl acetate to water). When the solvents were removed, the nanosheets aggregated automatically and then they were observed by SEM. (c, d) The nanosheets were prepared with the same method but without any self-assembly strategy. The results indicate that the self-assembly of the nanosheets in the liquid-liquid interface allows the nanosheets to be oriented in an ordered way. Scale bars: (a) 10 μm; (b) 10 μm; (c) 500 nm; (d) 10 μm.

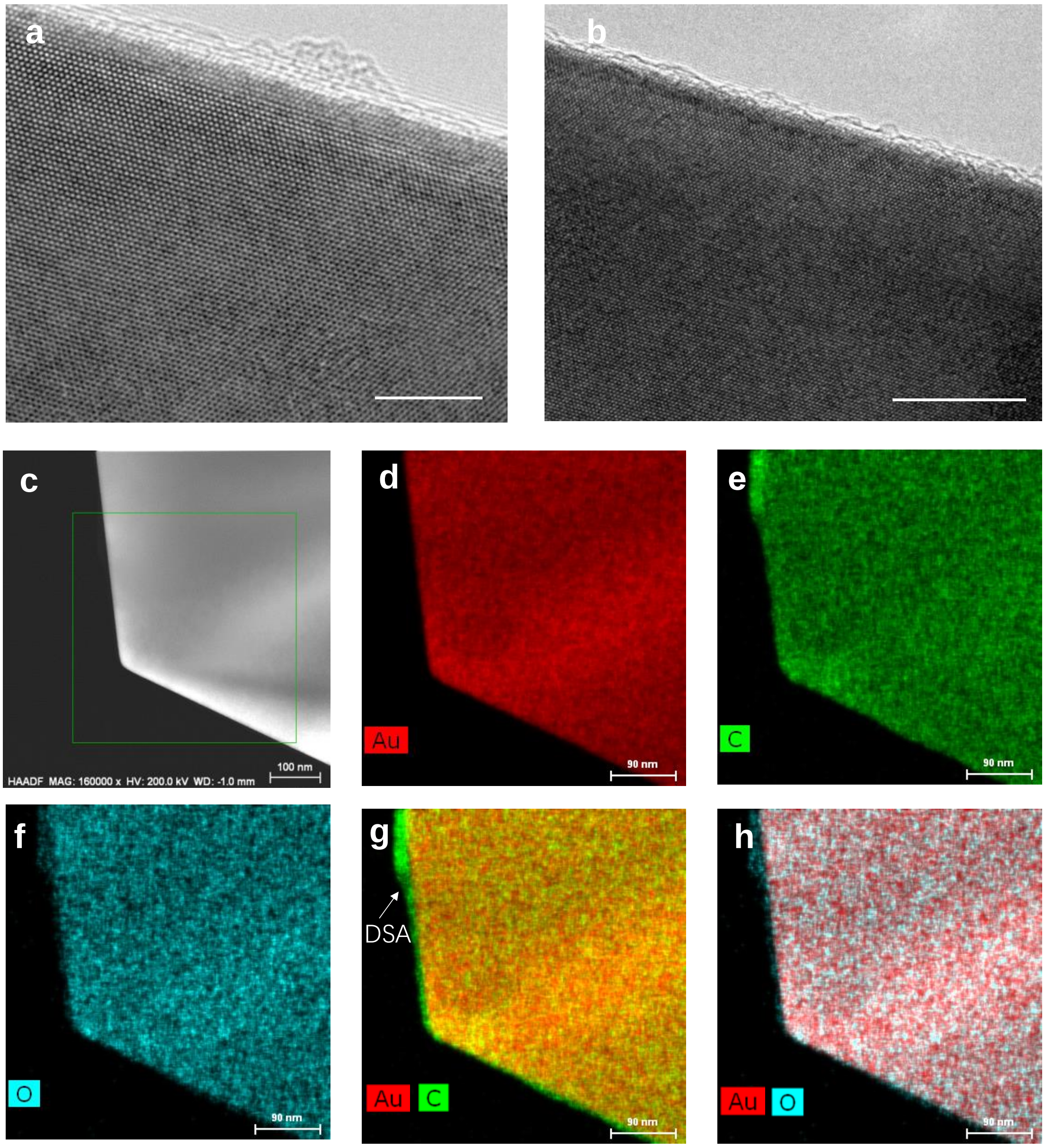

Supplementary Fig. 10 TEM and EDX images of a single-crystalline gold nanosheet. (a, b) High-resolution TEM images of the single-crystalline nanosheet. (c-h) EDX elemental mapping of a gold nanosheet. The images indicate that the surface of the nanosheet are adsorbed by DSA molecules. The ligand density of DSA at the surface of the nanoplate is ~ 0.18g m^{-2}. Scale bars: (a) 5 nm; (b) 10 nm; (c) 100 nm; (d-h) 90 nm.

a

Element	Series	Net	unn. C [wt.%]	norm. C [wt.%]	Atom. C [at.%]	Error (3 Sigma) [wt.%]
Gold	L-series	587337	97.35	97.35	69.56	29.28
Carbon	K-series	15591	2.44	2.44	28.62	0.30
Oxygen	K-series	3057	0.21	0.21	1.82	0.10

b

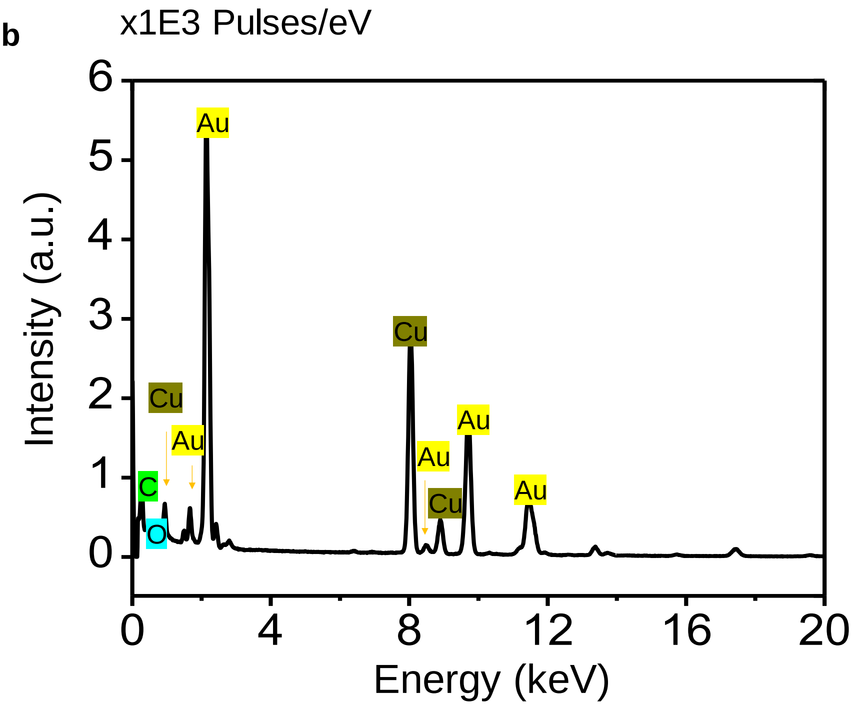

Supplementary Fig. 11 The EDX spectrum of a nanosheet on a copper support grid. Peaks for Au arise from nanosheets, and peaks for C, O from DSA molecules. (a) The estimated atom weight percentages from (b) the EDX mapping spectrum are Au:(C and O) = 97.3%:2.7%. The Cu signals were derived from the C/Cu TEM grid used for the analysis.

We found after three times of solvent extraction (repeatedly remove ethyl acetate and added new solvents), the DSA in the system become less (1.7 % weight loss) compared with original DSA percent, but not completely removed, which was characterized by TG-DTA of the gold clay at a 10 °C min^{-1} in nitrogen.

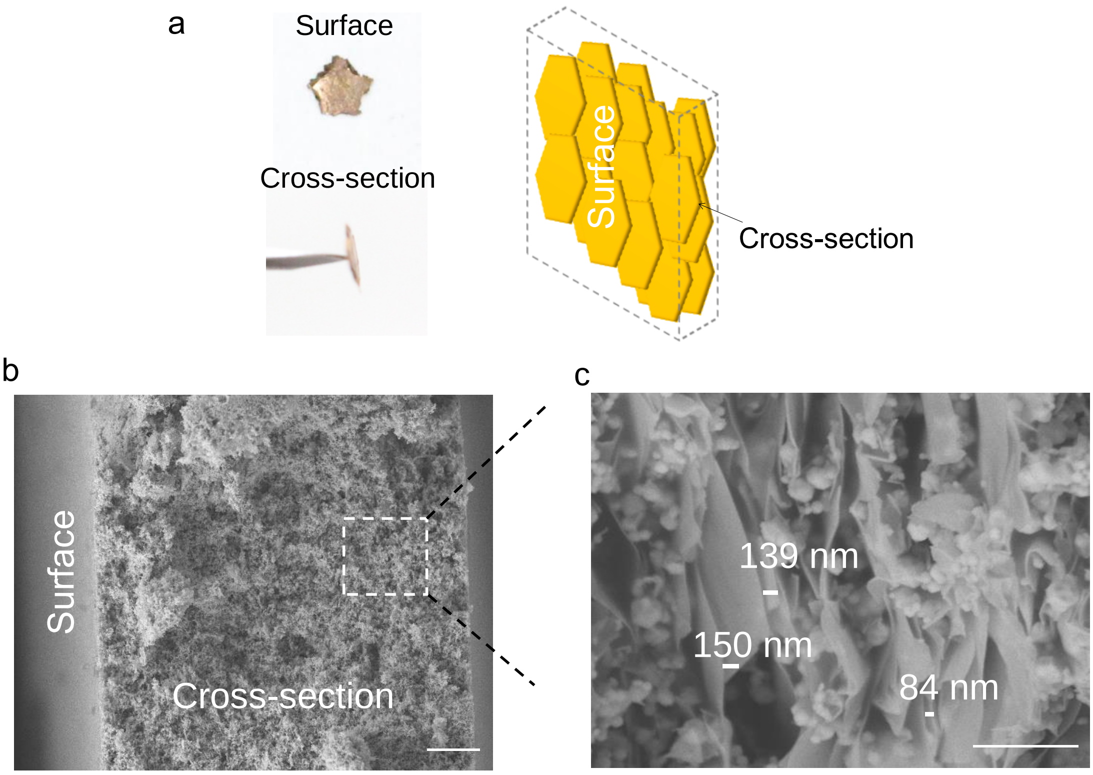

Supplementary Fig. 12 SEM characterization of a free-standing, thin gold film (thickness: 70 μm) in the cross-section after mechanical compression to a strain of ~60%. (a) Photo images and structural illustration of the thin gold film. (b, c) It shows a porous structure at different magnification in the cross-section with a decreased nanosheet interlayer distance.
Beside nanosheets, the presence of spherical particles may also improve the connectivity between the nanosheets upon compression. The total pore value of the gold clay before compression is ~0.002 cm^3 g^{-1}. The surface area of the gold clay before and after mechanical compression (60%) is measured to be 1.49 m^2 g^{-1} and 1.23 m^2 g^{-1}, respectively. Scale bars: (a) 10 μm; (b) 1.0 μm.

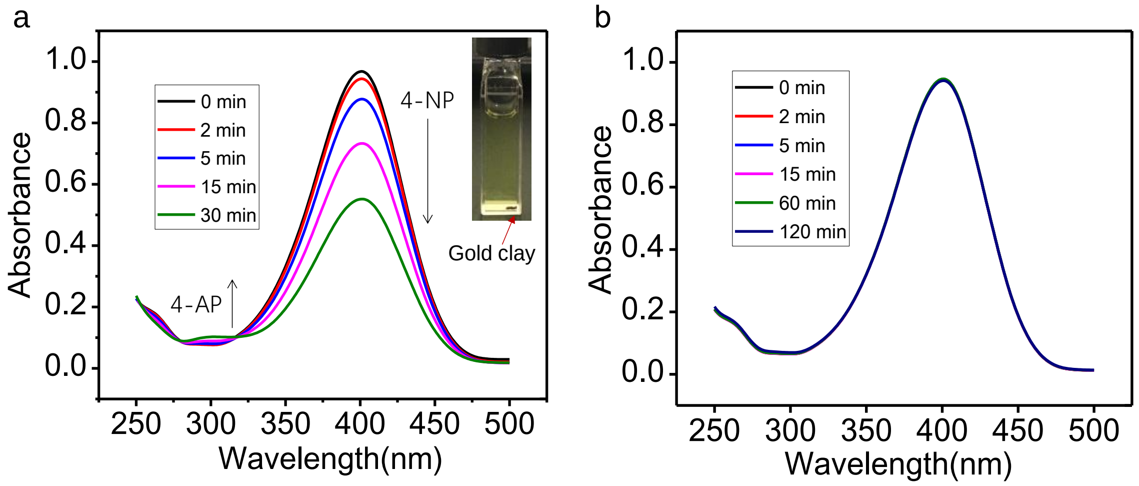

Supplementary Fig. 13 Catalytic performance of gold clay in the reaction of 4-nitrophenol (4-NP) reduction to 4-aminophenol (4-AP). The absorption spectra were recorded at different time in the reaction system: (a) in the presence of gold clay; (b) in the absence of gold clay. The gold clay was dried at 80 °C for 2 h to remove the solvents, and solvent extraction of the DSA by ethyl acetate for three times was performed before using. The catalytic reduction of 4-NP was carried out in a quartz cuvette. The freshly prepared aqueous solutions of $NaBH_4$ (20 mM, 2.94 mL) and 4-NP (15 mM, 30 μL) were mixed by addition of gold clay (1.2 mg) as catalysis. The solutions were mixed by shaking before each measurement. The reaction was monitored from 200 to 550 nm at different time intervals using UV–Vis absorption spectrophotometer.

The gold clay is in an aggregated state (fall at the bottom of the solution). The reactants (4-NP) need to diffuse from the bulk fluid phase to adsorb on the catalyst surface. Also, the adsorption site is not always an active catalytic site (covered by organic molecules), so reactant molecules must migrate across the surface to an active site. This affects the efficiency of the gold clay as catalysis. There is one advantage to use clay as catalysis due to the aggregated porous structure. The gold clay as catalysis can be recollected (separated) from the solution by a simple filtration and quickly reusable for the next reaction. In the case of a common noble metal nanoparticle (as catalysis), they are dispersed in the solution. The recycle of the nanoparticle from solution is difficult and other additional procedures such as loading nanoparticles in porous metal oxides (such as SiO_2) is required.

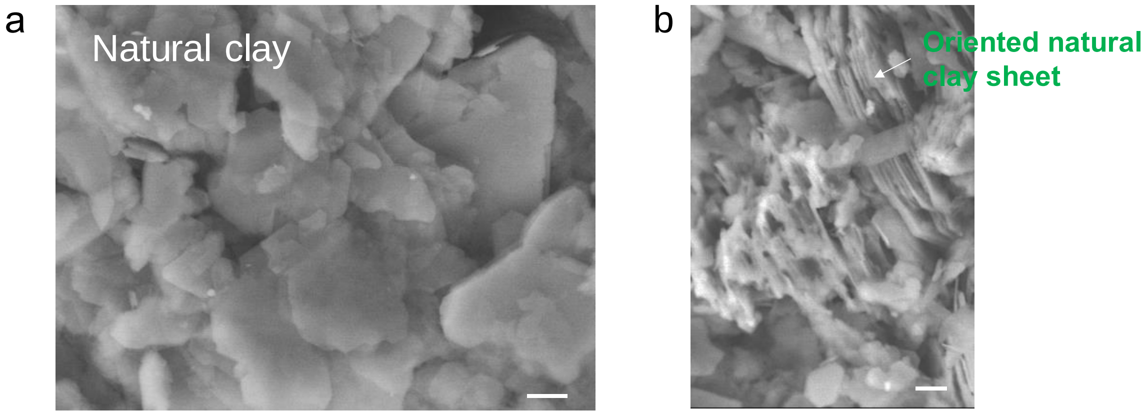

Supplementary Fig. 14 SEM image of a natural clay mineral (kaolinite). It can be found that there are a bunch of clay nanosheets that are oriented parallelly and stick to each other from (a) the surface and (b) cross-section images. Besides nanosheets, there are many particles.

The natural clay mineral (kaolinite, $Al_2O_3 \cdot 2SiO_2 \cdot 2H_2O$) was bought from Sigma-Aldrich and used as received. The SEM images was taken after drying the sample (a mixture of kaolinite with water) in an oven at 80 °C for two hours. Scale bars: 1.0 μm.

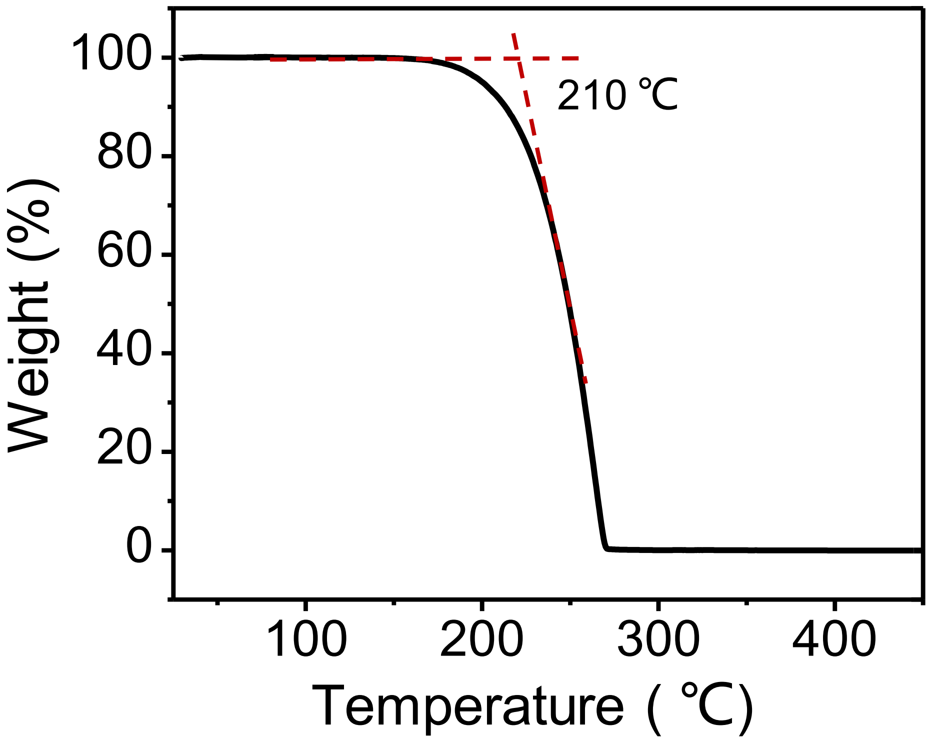

Supplementary Fig. 15 The thermogravimetric (TG) spectrum of the DSA compounds. The TG thermograms were recorded in the temperature range from 30 °C to 450 °C at a heating rate of 10 °C min^{-1} in nitrogen atmosphere.

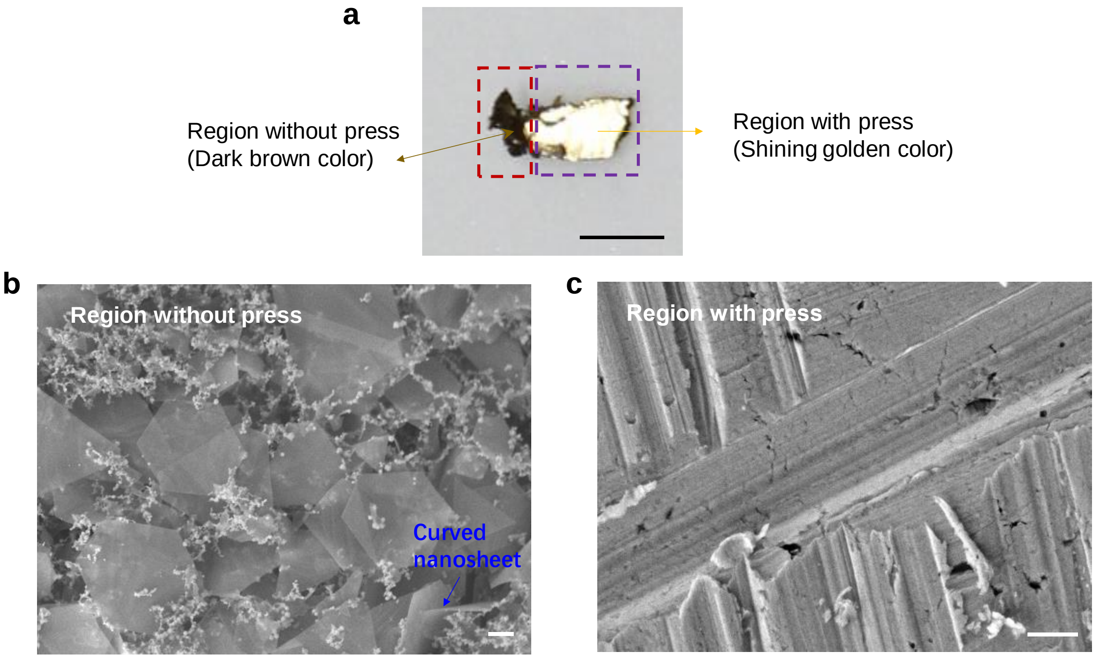

Supplementary Fig. 16 Surface morphologies of the film in the regions with and without compression. (a) The region of the film without compression (left) showed dark brown color with a structure of oriented gold nanosheet packing. (b) There was some curved nanosheet in the structure, indicating its softness. However, the region after compression (a, right) showed a shining golden color with a completely difference surface morphology. (c) The surface became fused and compact from SEM observation.

Due to the softness of these nanosheet assemblies, they can be easily compressed by hand at room temperature. Thus, it may supply a new bottom-up method to produce thin gold foils (for use in gilding etc.) in an easy and energy-saving way. Scale bars: (a) 1.0 cm; (b) 1.0 µm; (c) 1.0 µm.

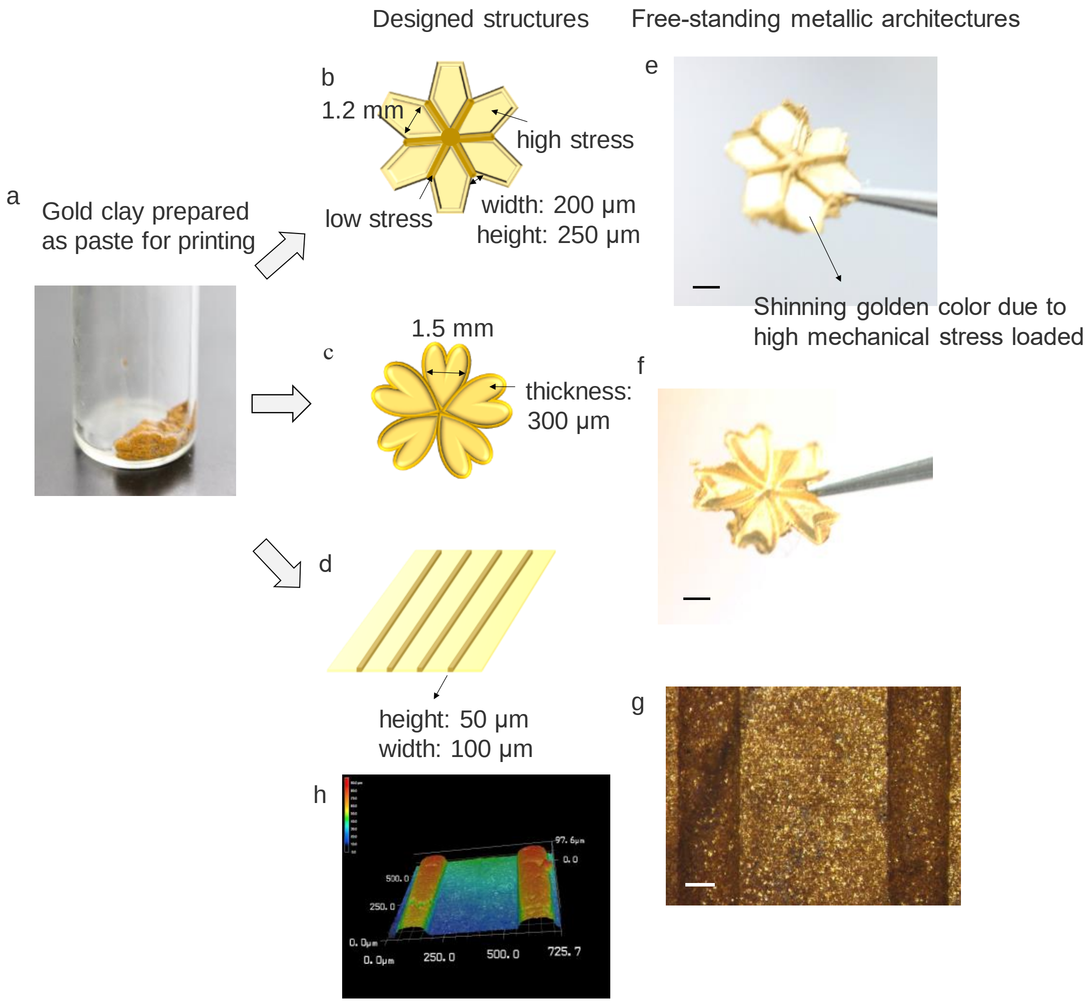

Supplementary Fig. 17 Free-standing metallic architectures using gold clay. The gold clay as a paste (a) can be printed into designed 3D structures (b-d) using moulds under compression by hand. The printed hybrid metallic architectures (e-f) show shining golden color and the printed stripe pattern (g-h) shows high resolution. Scale bars: (e) 1.0 mm; (f) 1.0 mm; (g) 50 μm.

Supplementary References

1. Huang, Y. *et al.* High-yield synthesis of triangular gold nanoplates with improved shape uniformity, tunable edge length anf thickness. *Nanoscale* **6**, 6469 (2014).

2. Miranda, A. *et al.* One-pot synthesis of triangular gold nanoplates allowing broad and fine tuning of edge length. *Nanoscale* **2**, 2209 (2010).

3. Ah, C. S., Size-controlled synthesis of machinable single crystalline gold nanoplates. *Chem. Mater.* **17**, 5558 (2005).

4. Liu, H., Yang, Q., A two-step temperature-raising process to gold nanoplates with optical and surface enhanced Raman spectrum properties. *CrystEngComm*, **13**, 2281 (2011).

5. Pienpinijtham, P. et al. Micrometer-sized gold nanoplates: starch-mediated photochemical reduction synthesis and possibility of application to tip-enhanced Raman scattering (TERS). *Phys. Chem. Chem. Phys.* **14**, 9636 (2012).

6. Xie, J., Lee, J. Y., Wang, D. I. C, Synthesis of Single-Crystalline Gold Nanoplates in Aqueous Solutions through Biomineralization by Serum Albumin Protein. *J. Phys. Chem. C.* **28**, 10226 (2007).
