# Supplementary material for: Gold clay from self-assembly of 2D microscale nanosheets
Source: Nat Commun. 2020 Jan 29;11:568. doi: 10.1038/s41467-019-14260-5 (PMC6989663; doi:10.1038/s41467-019-14260-5)
Supplement: Supplementary file 2 — Description of Additional Supplementary Files [file 41467_2019_14260_MOESM2_ESM.pdf]

Description of Additional Supplementary Files

File name: Supplementary Movie 1

Description: The synthesis of gold nanomaterials in DSA (C12) aqueous solution. The synthesis was simple as shown in the Movie. When keeping the reaction bottle in the water bath (53°C) without any stirring, a slightly red colour change was occurred at the water/air interface due to the localized surface plasmon resonance (LSPR), and then the colour spread to the bulk solution. (Total time: 12 min)

File name: Supplementary Movie 2

Description: Part 1. The self-assembly of the nanosheets at the liquid-liquid interface. After adding ethyl acetate to the synthesized nanosheets in water, they immediately aggregated at the interface especially after shaking. Part 2. Such self-assemble phenomenon was not happened in the case of small nanoparticles (25 nm) prepared with the same method.

File name: Supplementary Movie 3

Description: Part 1. The electrical conductivity measurements by using four-probe method. A commercial ITO glass showed a typical resistance of ~10 Ω sq-1, and the hand-deformed (pressed) gold clay showed a low resistance of ~7.7×10-3 Ω sq-1. Part 2. The gold clay was used as a conductive paste to write characters on paper.

File name: Supplementary Movie 4

Description: The free-standing metallic architecture produced by using gold clay as a paste upon compression without any thermal annealing. It showed shinning golden color due to the morphology change upon mechanical compression.
